# Editorial: Applied psychoanalysis and psychoanalytically informed research

**DOI:** 10.3389/fpsyg.2026.1956380

**Published:** 2026-09-02

**Authors:** David Titelman

**Affiliations:** Department of Learning, Informatics, Management and Ethics (LIME) National Center for Suicide Research and Prevention, Karolinska Institutet (KI), Solna, Sweden

**Keywords:** André Green, applied psychoanalysis, ecological crisis, Freud, metapsychology, psychoanalytic listening, transference, universal symbols

## What is applied psychoanalysis?

In her contribution to this volume—a review of the vicissitudes psychoanalysis after World War 2 and a discussion of psychoanalytic research in our times—the co-editor of this volume, Leuzinger-Bohleber, writes that “psychoanalysis understands itself as a science of the unconscious and has developed specific methods for exploring unconscious fantasies and conflicts.” The first part of this statement—that the primary empirical field of psychoanalysis is unconscious affect and thought—was indeed my starting point when, having accepted the invitation to propose a psychoanalytic Research Topic in Frontiers, I asked professors Leuzinger-Bohleber^1^ and Henrik Enckell^2^ to join me in this challenge. The three of us share the experience that psychological research is enriched by including clinically well-grounded psychoanalytical hypotheses and methods; we also share a wish to promote the standing of psychoanalysis as a scientific discipline in the larger academic environment.

The second part of Leuzinger-Bohleber's formulation is methodological: How can unconscious mental phenomena be studied? A simple answer to this question could be by listening psychoanalytically to the patient or research subject. In my review of Freud and Judaism in this volume, I studied Freud's relationship to his father by rereading and listening not only to the printed narrative of his penultimate *Moses and Monotheism* (Freud, 1939), but also to how this text affected me as a reader. A third inroad of this inquiry (or aspect of my listening) was of course to read the relevant biographical and historical material at hand.

A researcher's identification with his or her study object (in my case, Freud, including *his* identification with Moses) is analogous to the *countertransference*, the analyst's disciplined libidinal investment of the patient that may evolve in a psychoanalytic treatment relationship. Strong emotions evoked in the observer—not only in psychoanalysts and other care professionals, but also in empirical researchers in relation to their study objects—may have dire consequences. However, much can be learned from reflecting on such feelings, including about the unconscious wishes and motivations of the studied other. In their study in this issue of the effects of a psychotherapeutic intervention supporting socially disadvantaged pregnant women at risk for perinatal depression, Gomà et al. included the scrutiny of their observers' countertransference as a source of information about their research subjects.

Is, then, the reflection on the observer's transference onto his or her study object -be this object a person, a clinical or otherwise vulnerable group, or a research question- a defining quality of applied psychoanalytic studies? This dimension of the empirical field was considered in several of our contributions, even when it wasn't explicitly addressed by the author, for example, in the study by Taipale on projective identification, the study by Goldreich on the use and users of psychedelics (in which the introduction of Matte Blanco's bi-logic model of psychic life as supplement to Freud's metapsychology—his never-completed psychology of the unconscious—is an added benefit), the study by Lawrence Fishman who in his dense meditation adds Lacanian perspectives to Freud's theory in elucidating Marcel Proust's self-healing through writing, and in the interdisciplinary study by Ambresin et al. on trauma, dreams, and memory in a psychoanalytic patient. Hoff's and Strømme's study on smiles and laughter in different forms of psychotherapy can be included here, albeit that these investigators, in the name of scientific rigor, chose not to analyze the fluctuating attunement between therapist and patient in psychoanalytic terms. This ambition is not unique. (Green 2005) has noted that rendering psychoanalytic lines of thought as scientific formulations other than those of Freudian metapsychology and clinical theory may protect the writer from the accusation of being unscientific. He claimed that such translations are a recurring feature of applied psychoanalysis.

Another subgroup of the studies, links to psychoanalysis in another way. The contributions by Meurs et al. on the “pretend mode” in mentalization-based psychotherapy with adopted children and children in foster care, that by Schestag et al. on migrant and exiled mothers and their infants, and the already mentioned study by Gomà et al. all aspire to statistically analyze quantitative measures of psychoanalytically relevant questions notwithstanding that these questions by definition are qualitative. Authors of such studies often conclude their discussions by recognizing a need to supplement their findings either by more extensive work or by in-depth qualitative studies in their respective fields.

There is a noticeable symmetry between this slightly apologetic attitude (preferable to its conceivable self-satisfied opposite) and that of Freud, who throughout his work intermittently vented his frustration over what he eventually called his “witch” metapsychology (Freud, 1937, p. 225), again, his theoretical corpus that he revised and expanded until the end; Freud loathed eliminating formulations once put in print. (This attitude is suggestively similar to the Talmud in which one rabbi's argument against another's does not lead to the elimination of the view just refuted; both opinions remain in the canon.) From its inception (Freud 1900) saw his theory as a temporary “scaffolding” (p. 610) that compensated for the lack of means for studying the assumed biological substratum of human psychology, which, as mentioned by Ambresin et al., he had set out to uncover in 1895 (Freud, 1895).

As the grand theory of psychoanalysis may thus seem overwrought with competing or sometimes contrary points of view, it is not surprising that critics, including serious students, find issues to discuss. In this volume, Mellor, for example, suggests replacing the terms *death drive* and *Eros* with the binding and unbinding of Fristonian *free energy*, which concept, Mellor seems to maintain, less ambiguously lends itself to describing what he calls “the vital rhythms of life and self-organization.” Karlsson's contribution is a personal wrestling with oft-debated issues such as phallic monism, sexuality and gender in infant care, and the caring dimension or lack thereof in psychoanalytic therapy. To find a resolution of his quandaries, at least those about psychotherapy, Karlsson turns to Martin Buber's *I-Thou* perspective as an ideal for a psychotherapeutic relationship, which he believes is less reifying than that of the psychoanalytic (conflict-interpreting and transference-focused) method. And in their concentrated review of the psychoanalytic debates on of symbolism in scientific discourse—from the *Interpretation of Dreams* (Freud, 1900) to (Enckell 2010) —Huo and Ju critically discuss Freud's use of symbols, particularly his idea of universal symbols. They nonetheless conclude that discarding the exploration of Freudian universal symbols, including the phylogenetic aspect of unconscious wishes and fantasies that Freud for long withheld from publication (cf. Freud, 1987) but embraced toward the end of his life, would mean “forfeiting a scientific dialogue dedicated to the universality of the human mind.”

In contrast to these critiques (yet in agreement with the conclusion by Huo and Ju), Lehtonen and Lehtonen's contribution is an analysis of the impact and faulty containment of the real existential threat that the ecological crisis of our times amounts to. They scrutinize the widespread denial of the problems the world faces through the theoretical lenses of (a) Freud, who noted a potentially creative, playful composure as a prerequisite of psychic survival and mature action; (b) Winnicott, whose work can inspire a mature response to fear-of-breakdown-fantasies, which the authors emphasize are universal; (c) Bion, who addressed the capacity to think under existential threat; and (d) a selection of findings in contemporary neuroscience that substantiate the psychoanalytic inroads to preserving one's sanity and judgment in times of crisis. The presented theory is brilliantly integrated and the nature of the analysis close to what may be the original model of applied psychoanalytic social studies: that of Freud struggling to understand his increasingly self-destructive society and its mores in works such as *Civilization and its Discontents* (Freud, 1930) and his book on Moses (Freud, 1939).

## Footnotes

1 Senior research fellow at the university clinic of Mainz and former director of the Sigmund Freud Institute in Frankfurt, Germany.

2 Associate professor at Jyväskylä University, Finland, and training analyst at the Finnish Psychoanalytic Society.
